# Resistance exercise and Alzheimer’s disease blood‐based biomarkers in European cognitively healthy older adults

**DOI:** 10.1002/alz.088043

**Published:** 2025-01-09

**Authors:** Irene Esteban‐Cornejo

**Affiliations:** ^1^ Instituto de Investigación Biosanitaria (ibs.GRANADA), Granada, Spain; Faculty of Sport Sciences, Sport and Health University Research Institute (iMUDS), University of Granada., Granada, Granada, Spain; CIBER de Fisiopatología de la Obesidad y Nutrición (CIBEROBN), Instituto de Salud Carlos III, Granada Spain

## Abstract

In the quest to combat Alzheimer’s disease (AD), physical exercise has emerged as one of the leading non‐pharmaceutical approaches to improve executive function and ameliorate cognitive decline. The limited understanding of the mechanisms by which exercise impacts cognition in late adulthood hinders the widespread use of exercise as a therapeutic or preventive approach for AD. This underscores the urgent need for research aimed at unraveling the mechanisms by which exercise improves cognition.

This study delves into the unexplored realm of AD blood‐based biomarkers as potential mediators of the cognitive benefits induced by exercise. Despite their potential significance, no previous trials have examined the impact of exercise on these novel AD BBMs and their cognitive implications. These effects may vary based on exercise type (e.g., aerobic, high intensity interval training or mind‐body exercises), necessitating a thorough exploration of optimal exercise types and doses before advancing to comprehensive interventions.

Using data from the AGUEDA trial, a single‐site, two‐arm, single‐blinded, randomized controlled trial (RCT) involving 90 cognitively normal older adults from Spain (Europe), aged 65‐80 years old, this study marks the first attempt to uncover the effects of a 24‐week resistance exercise intervention on AD BBMs and their cognitive implications. We will share promising findings on the impact of exercise on AD BBMs related to AD pathological hallmarks (i.e., amyloid‐β peptides and p‐tau isoforms), to neuronal loss (NfL); and to neuroinflammation (GFAP).

This research not only will unveil the potential of resistance exercise as a powerful tool in AD prevention but also will help to set the stage for a new era of precision interventions targeting specific AD BBMs.

